# Observation of synthetic moving effect in metamaterials

**DOI:** 10.1038/s41377-026-02361-y

**Published:** 2026-06-08

**Authors:** Qingdong Yang, Zhongfu Li, Xinhua Wen, Oubo You, Teruya Ishihara, Xiaobo Yin, Shuang Zhang

**Affiliations:** 1 New Cornerstone Science Laboratory, Department of Physics, University of Hong Kong, Hong Kong, China; 2 Department of Mechanical Engineering, University of Hong Kong, Hong Kong, China; 3 State Key Laboratory of Optical Quantum Materials, University of Hong Kong, Hong Kong, China; 4 Materials Innovation Institute for Life Sciences and Energy (MILES), HKU-SIRI, Shenzhen, China; 5 Quantum Science Center of Guangdong-Hong Kong-Macao Greater Bay Area, 3 Binlang Road, Shenzhen, China; 6 Department of Electrical and Electronic Engineering, The University of Hong Kong, Hong Kong, China

**Keywords:** Metamaterials, Magneto-optics

## Abstract

When light propagates through a flowing transparent fluid, the refractive index it experiences depends on its propagation direction relative to the flow direction. This leads to different phase delays for light traveling along or against the flow. The moving effect, which results from the cross-coupling between electric and magnetic responses in a specific way, is only detectable over a considerable distance within the moving medium, because the fluid’s velocity is much smaller than the speed of light *c* in realistic systems. In the context of metamaterials – artificial subwavelength structures – it may be possible to manipulate electromagnetic fields to mimic the moving medium without physical motion. Here we demonstrate a passive metamaterial that achieves a significant synthetic moving effect. The metamaterial is composed of artificial metallic structures and gyromagnetic materials, and is meticulously designed based on symmetry and electromagnetic considerations to achieve significant pure bianisotropic moving coupling. Our experiment reveals for the first time a gigantic moving response of the metamaterial with an effective velocity reaching 0.3c. The inherent strong non-reciprocity of this moving response opens the door to a variety of photonic devices, like polarization-independent gyrators and isolators.

## Abstract (web summary)

A passive metamaterial achieves a synthetic moving effect with effective velocity up to 0.3c, enabling polarization-independent nonreciprocal transmission and isolators without physical motion.

## Graphical abstract

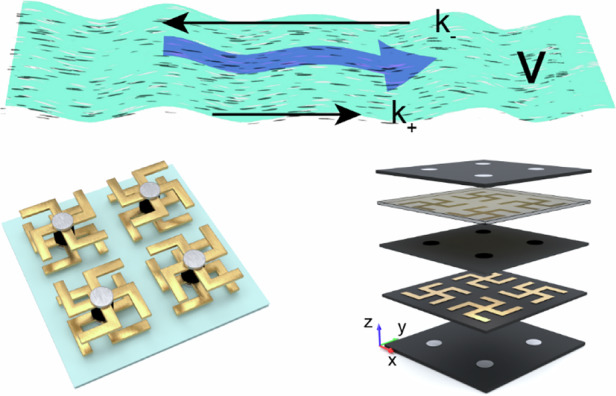

## Introduction

**Fig. 1:**
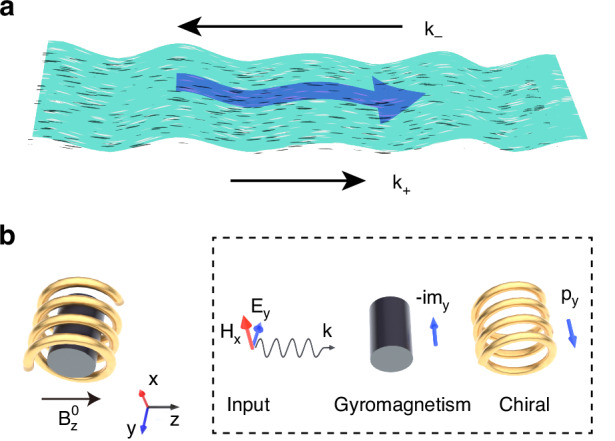
Mechanism of metamolecules for achieving moving response. **a** Optical response of a moving fluid. Wavevector and hence the light speed (refractive index) depends on the propagation direction of light. Specifically, light propagating along the fluid flowing direction exhibits a wave vector less than that against the flow. **b** A metamolecule design for achieving moving effect. The meta-molecule consists of a YIG rod biased by an external static magnetic field ${B}_{z}^{0}$ embedded inside a chiral structure. The *x*-oriented magnetic field *H*_*x*_ of the incident wave induces a magnetic dipole -*im*_*y*_ and then excites the electric current along the chiral structure inducing the electric dipole *p*_*y*_

Light speed in flowing water depends on the velocity of the flow relative to the light, which was experimentally observed by Fizeau in 1851^1^ using an interferometric setup. Although the experiment initially aimed to detect the light-dragging effect of ether, later it turned out that the observed results were unrelated to mechanical drag. Instead, the velocity addition law in the theory of special relativity can properly explain the observed results^2–4^. According to the special relativity, the constitutive relations for moving medium along *z*-axis are expressed as^5^

1 $$\begin{array}{c}{\boldsymbol{D}}=\varepsilon {\varepsilon }_{0}{\boldsymbol{E}}+\frac{\xi }{c}\bar{{\boldsymbol{z}}}\times {\boldsymbol{H}}\\ {\boldsymbol{B}}=\mu {\mu }_{0}{\boldsymbol{H}}-\frac{\xi }{c}\bar{{\boldsymbol{z}}}\times {\boldsymbol{E}}\end{array}$$

where $\xi =\frac{v}{c}\frac{{{n}^{{\prime} }}^{2}-1}{1-{(v/c)}^{2}{{n}^{{\prime} }}^{2}}$ is the magnetoelectric coupling term, *c* and *v* are the velocity of the light and fluid, respectively, and *n*' is the refractive index of the medium at rest. Here we assume that light is propagating along *z*-axis and $\bar{{\boldsymbol{z}}}$ is its unit vector. *ε* and *μ* are permittivity and permeability of the moving medium, respectively. Thus, in moving media, the electric displacement field ***D*** is induced not only by the electric field ***E*** but also by the magnetic field ***H*** in a specific way. Generally, cross coupling of electric and magnetic fields in the constitution equation is referred to as bianisotropic coupling^6,7^. There are various types of bianisotropic coupling, including reciprocal chiral^8,9^ and Omega^10,11^ coupling, as well as nonreciprocal Tellegen^12–14^ and moving^15^ coupling. Notably, the bianisotropic moving coupling features real-value cross-coupling between the electric and magnetic responses along orthogonal directions, as indicated by *ξ* in Eq. (1). This moving response breaks time-reversal symmetry and results in a nonreciprocal response^16,17^. As a result, the refractive indices for forward and backward propagating waves are different, as shown in Fig. 1a. However, the difference is very small in realistic systems because the achievable velocity for fluid is much smaller than the light speed in the medium. Specifically, the forward and backward propagation of light inside the moving materials has an effective index of ${n}_{+}=\sqrt{\varepsilon \mu }-\xi$ and ${n}_{-}=\sqrt{\varepsilon \mu }+\xi$, respectively. The derivation of the refractive index for a general constitutive relation is provided in Supplementary Materials Sec. 1.

Given the difficulties in obtaining media with significant moving response, it is proposed to employ artificially designed structures^18–20^, without any physical motion to emulate the behavior of electromagnetic waves in media in motion, which would set free moving related fundamental studies and applications from the fluid medium^21–24^. Metamaterials, composed of sub-wavelength artificial structures, offer the advantage of being easily customizable to achieve extraordinary electromagnetic responses, such as negative refractive index, cloaking media, ultrathin flat lens, and artificial gauge field^25–28^. In addition to enabling engineering the permittivity *ε* and permeability *μ*, metamaterials can also be designed to control different types of bianisotropic coupling. Despite the great interest in achieving a synthetic moving effect with metamaterials at rest^29^, the experimental demonstration of significant pure moving response has remained elusive. Previous experimental endeavors have involved employing the transmission line to mimic the behavior of electromagnetic waves in moving media in megahertz, while the achieved effective velocity has been two orders of magnitude smaller than the speed of light in vacuum. In addition, in the practical realization of the transmission line, imperfect coupling between the inductors and parasitic capacitance and inductance disrupt the pure bianisotropic moving response, and stability conditions need to be considered due to the introduction of active elements^18^.

Here we present the experimental demonstration of metamaterial for achieving pure bianisotropic moving response based on symmetry and electromagnetic consideration and experimentally demonstrate a gigantic moving effect at microwave frequencies. We use gyromagnetic material and chiral structure to design the synthetic moving metamaterials with in-phase magnetoelectric coupling. When a wave with arbitrary polarization impinges on the slabs from the opposite sides, we show that the amplitudes of transmission are identical, but their phases are different. Meanwhile, the reflection remains the same from both incident directions. These are the unique features to distinguish the bianisotropic moving coupling from other bianisotropic couplings. Importantly, an effective moving speed as large as 0.3c, comparable to the speed of light, is demonstrated in our experiment, paving the way towards new schemes for designing non-reciprocal photonic devices, like polarization-independent isolators and gyrators.

## Results

### Artificial moving metamaterial design

To design an artificial moving response, it is essential to break the time-reversal symmetry. Here we will focus solely on passive systems. At microwave frequencies, the most viable options are gyroelectric and gyromagnetic materials (GM). While incorporating gyroelectric materials such as magnetized plasma proves challenging, gyromagnetic materials such as Yttrium-iron-garnet (YIG) can be readily utilized to introduce strong gyromagnetic responses with very low loss^30,31^. To transform the gyromagnetic response into the moving response, which entails real-valued cross-coupling (i.e., in-phase cross-coupling) between the electric and magnetic responses along orthogonal directions, one can combine nonreciprocal gyromagnetic response with reciprocal bianisotropic responses, i.e. chiral and Omega effects. Interestingly, the desired in-phase coupling of moving response can be achieved by the combination of *π*/2 phase delays from both gyromagnetic and bianisotropic effects. Figure 1b illustrates one possible meta-structure configuration to realize the electromagnetic moving response. It consists of a chiral structure and a GM biased by a static magnetic field ${B}_{z}^{0}$ along the *z*-direction. When a beam propagating along the *z-*direction with an *x*-oriented magnetic field *H*_*x*_ impinges on the GM, *H*_*x*_ induces a magnetic dipole moment -*im*_y_ due to the gyromagnetic effect (i.e., the antisymmetric elements of the GM permeability tensor), where the imaginary unit *i* stands for *π*/2 phase shift between magnetic dipole and the magnetic field *H*_*x*_. Through electromagnetic induction, the time-varying magnetic dipole field excites an electric current in the chiral structures, leading to accumulated charges of opposite signs on two ends of the chiral structure, which form an electric dipole along the *y*-direction. By combining the -*π*/2 phase delay from the gyromagnetic effect and the *π*/2 phase delay caused by chiral structures, the resulting *p*_*y*_ is in phase with the incident magnetic field *H*_*x*_, exactly achieving the desired bianisotropic moving coupling.

**Fig. 2:**
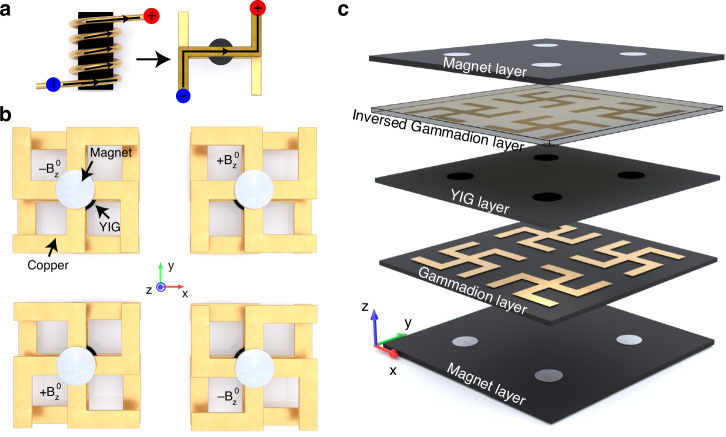
Design of planar metamaterials with moving effect. **a** Deformation of the original design of Fig. 1b to layered geometry which is part of up-right part of **b**. The dark and light yellow represent the upper and lower metal respectively. The accumulated charge and current are similar to each other which effectively generates the moving response. **b** The configuration of the unit cell of a metamaterial consists of four metamolecules. Direction of local static magnetic field at YIG (black cylinder) is shown in the center of the gammadions (yellow color), which is flipped in accordance of direction of the gammadion so that the moving effect of these meta-molecules adds up, while the gyromagnetic and chiral response vanishes. The unit cell structure has a periodicity of 14.8 mm in the x–y plane and a thickness of 3 mm. **c** A decomposition of the actual unit cell, with multiple layers, where silver and black cylinders representing the magnets and YIG rods respectively are placed inside PCB slabs. The gammadion layer and the inversed gammadion layers are formed as a chiral structure, where inversed one at the bottom of the upper PCB slab is shown transparently

Based on practical fabrication considerations, the original structure in the left panel of Fig. 1b is modified to a pair of crank structures sandwiching a GM in Fig. 2a. The double crank structure can also provide a strong chiral response. The accumulated charge and current are shown in the structure caused by the gyromagnetic effect under a *H*_*x*_ polarized incident wave. In order to obtain in-plane isotropic response, we overlap the 90-degree rotated crank structures to form a pair of conjugated gammadion structures (upper right part of Fig. 2b), which has been widely exploited to realize strong isotropic chirality in the *x-y* plane^8,32–34^. Thus, isotropic moving response would be realized in a layered structure, which is much easier to fabricate than the original design. In addition, the resonating conjugated gammadion structure enables a strong confinement of the magnetic field within the gap of the two layers, thus the incident field can efficiently interact with the YIG material placed inside the gap, resulting in a notably enhanced bianisotropic moving response (see more details in Supplementary Materials Fig. S1). However, in addition to the moving response, these metamolecules also have other unwanted parasitic effects such as chiral and gyromagnetic effects. By reviewing the moving mechanism, we notice that the moving response changes its sign by reversing each of two factors, chirality and the direction of bias magnetization, which are responsible for chiral and gyromagnetic responses, respectively, while the sign is preserved when both factors are simultaneously reversed. To eliminate other parasitic effects, we construct a supercell consisting of two types of metamolecules: the first, located in the upper‑right and lower‑left of Fig. 2b, and its inverse counterpart, positioned in the lower‑right and upper‑left, with both chirality and bias magnetic field reversed. This configuration effectively cancels chiral and gyromagnetic responses. Meanwhile, this design, consisting of YIG rods under opposite magnetic fields and metallic structures of opposite chiralities, holds mirror symmetry with respect to the *x/y* axis (M_x_/M_y_) and parity–time (PT) symmetry, effectively excludes out unwanted responses but maintaining a strong moving effect (details in Supplementary Materials Sec. 3). We have noticed that the similar discussions of cancelling other bianisotropic effects are discussed in ref. ^35^ during the preparation of our paper. An alternative approach to synthetic moving metamaterial design, which combines gyromagnetic effect and Omega response, is elucidated in detail in Supplementary Materials Sec. 4. For a comprehensive understanding of these two design approaches, more details regarding the dipole moments induced by the moving response are provided in Supplementary Materials Sec. 5.

### Experiment measurement

**Fig. 3:**
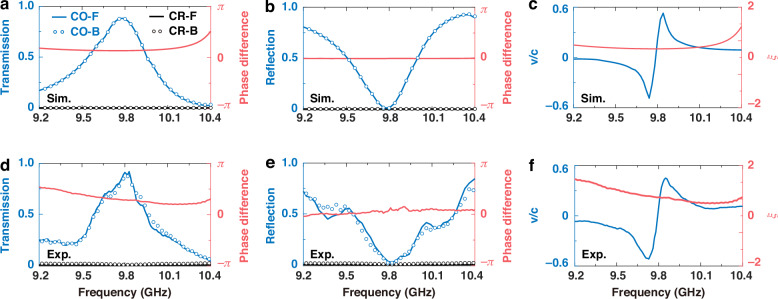
Experimental demonstration of moving effect. **a**, **b** Simulated and **d**, **e** measured transmission and reflection spectra when E_*y*_ incident wave. The labels “CO-” and “CR-” indicate co-polarization and cross-polarization respectively, and “F” and “B” represent forward and backward incident directions respectively. The phase difference between transmitted waves propagating in opposite directions is depicted by red lines in **a**, **b**, demonstrating that the moving response persists across the entire frequency range of interest. Simulated **c** and experimental **f** extracted effective artificial moving coupling parameter *ξ* and effective velocity *v*

To experimentally study the metamaterial with pure bianisotropic moving coupling, we fabricate a metamaterial following the design shown in Fig. 2b by employing the Printed Circuit Board (PCB) technology on a Teflon, which has a relative permittivity of 2.5. The configurations of the unit cell, decomposed into different layers, are illustrated in Fig. 2c. More details about the sample are provided in the Supplementary Materials Sec. 6. To unveil the nonreciprocity of artificial moving response, we derive the transmission of the artificial moving slab from opposite sides, which is given by

2 $${T}_{\pm }=\frac{4Z{{\rm{e}}}^{{\rm{i}}n{k}_{0}{d}_{0}}{{\rm{e}}}^{\mp {\rm{i}}{k}_{0}{d}_{0}\xi }}{{\left(1+Z\right)}^{2}-{{\rm{e}}}^{2{\rm{i}}n{k}_{0}{d}_{0}}{\left(1-Z\right)}^{2}}$$

where *T*_±_ is the transmission from the opposite sides, $n=\sqrt{\epsilon \mu }$ is the effective refractive index and $Z=\sqrt{\mu /\epsilon }$ is the impedance of the metamaterial. *k*_0_ is the vacuum wavevector and *d*_0_ is the effective thickness of the slab. For a lossless system, the transmissions in opposite directions have the same amplitude but with a phase difference 2*ξk*_0_*d*_0_. In contrast, the reflections are the same in both phase and amplitude. This feature is different from all other bianisotropic responses. The transmission and reflection spectra of the metamaterial, simulated using the commercial software COMSOL Multiphysics, are provided in Fig. 3a, b for both forward and backward directions under an *E*_*y*_-polarized incident wave. It is observed that the amplitudes of transmission in the forward and backward directions are indeed the same across the entire frequency range. A difference in the phase of the transmission between the opposite directions exists across a broad spectral range, proving the nonreciprocity of the designed metamaterials. Meanwhile, the amplitude and phase are identical for the reflections from both directions. There is no cross-polarization in both transmission and reflection for both incident directions. These features are characteristic of a moving response.

To characterize the designed sample, it was placed midway between two identical rectangular linearly polarized wideband horn antennas connected to the ports of a Vector Network Analyzer (VNA). Further details regarding the experimental measurements are provided in the Methods section. The measured transmission and reflection spectra of the metamaterial are shown in Fig. 3d, e, respectively. Our measurements verify the prominent transmission resonance at 9.8 GHz predicted by the simulation, which is attributed to the structural resonance of the conjugated gammadion. As predicted by theory, cross-polarized transmission is negligible for both simulation and experiment. The measured transmission and reflection spectra show good consistency with the simulated results in Fig. 3a, b. The slight difference in transmission amplitudes between forward and backward directions can be attributed to the optical loss in the metamaterial. The measured transmission phases are found to be significantly different for waves incident from the opposite sides, indicating the moving response in our structure. We noted that the slight discrepancy between the simulated and experimentally measured phase difference in our frequency range of interest likely stems from the assumptions made about the static magnetic field inside YIG rods. The simulation assumes a uniform field, whereas in practice, the field within the YIG rods may not be perfectly uniform, potentially affecting the measured response and accounting for the observed deviation. From the measured reflection, we also observe nearly identical amplitudes and phases from both incident directions. Similar to the simulated results, no cross-polarized component is observed in both transmission and reflection. Thus, the experiment results verify the existence of the moving responses in our structures. It is noteworthy that the metamaterial exhibits an in-plane isotropic moving response, thereby enabling polarization-independent nonreciprocal transmission. This property has been experimentally verified using various polarized incidence waves, such as linear polarized incident waves at 45 degrees and 0 degrees (x-polarized), as demonstrated in Supplementary Materials Fig. S6. The current metamaterial design employs resonant gammadion structures, which inherently restrict the transmission bandwidth. However, broadband performance can be achieved by designing broadband chiral structures (see Supplementary Materials Sec. 7).

Furthermore, we can extract effective moving parameters of the metamaterial by solving Fresnel’s equation using the transmission and reflectance spectra, and then obtain the effective velocity by employing the Lorentz transformation (refer to Supplementary Materials Sec. 8). Figure 3c, f present the retrieved moving coupling parameter *ξ* (red curves) and the corresponding effective velocity (blue curves). It should be noted that in Fig. 3c, f, only the real part of *ξ* is presented for clarity, although a small imaginary component exists due to the material loss inherent in the metamaterial. The extracted imaginary part of *ξ* and the effective refractive index *n* are provided in Supplementary Materials Fig. S9. Further discussion on the extracted effective velocity, which emulates the behavior of a physically moving medium, is provided in Supplementary Materials Sec. 9. Away from the resonance frequency, the velocity persists at a relatively substantial level (around 0.3c), while the imaginary component of the velocity becomes negligible (refer to Supplementary Materials Fig. S11). It is important to emphasize that such a significant velocity is unattainable in real moving media, allowing the metamaterial to emulate electromagnetic phenomena for media moving with a large velocity. In practical systems, the achievable effective velocity of the metamaterial is jointly determined by the chirality of the resonant metal structure and the gyromagnetic properties of the YIG rods, with optimal resonance conditions and strong coupling between these components being essential for its enhancement.

### Wavefront measurement

**Fig. 4:**
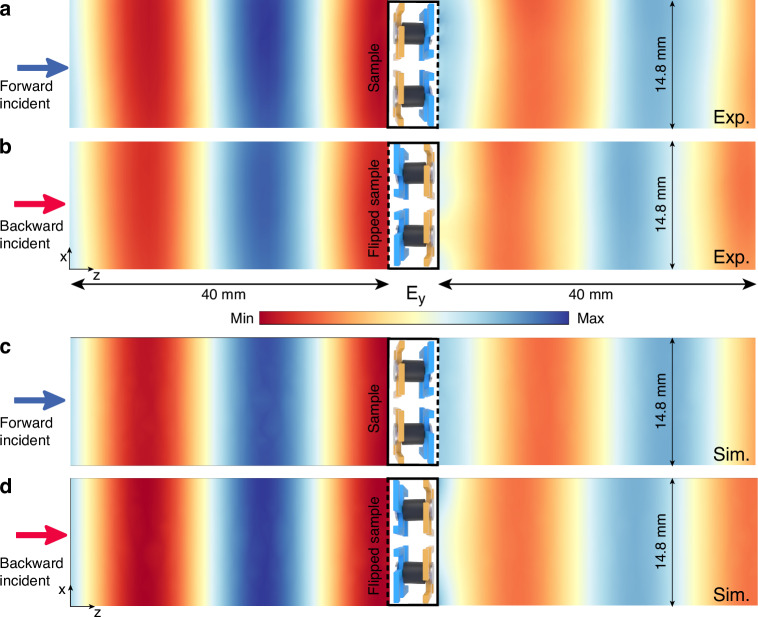
Wavefront characterization of the metamaterials slab. **a**, **b** Measured and **c**, **d** simulated *E*_*y*_ component of the wavefront in the *x*-*z* plane at 10 GHz. The +*z* incident wave is shown in **a**, **c**. For convenience of comparison, we keep the incident direction the same but flip the sides of the structure where -*z* incident wave is shown in **b**, **d**. The transmission side and reflection side are 5 mm and 15 mm above the slab respectively. The phase difference is observed in the transmission and the phase of reflection is the same from opposite incident directions. The amplitude is nearly identical from both incident directions

We further study the nonreciprocal response of the synthetic moving metamaterial via the measurement of the wavefront of the wave across the slab. Figure 4 shows the experimentally observed *E*_*y*_ component of the field distributions on the *x-z* plane of the sample from opposite incident directions at the frequency of 10 GHz. For convenience of comparison, we keep the incident direction the same but flip the sides of the structure, corresponding to opposite effective moving velocities of the medium. In the measured field distribution presented in Fig. 4a, b, appreciable reflection is observed, leading to wave interference on the reflected side and a reduction in transmission amplitude on the transmitted side. The field distribution on the reflection side appears the same in both cases, indicating the same reflection from opposite incident directions. In addition, the measured transmission amplitude almost remains the same for both cases. However, there is a noticeable phase difference of about 0.27*π* between the transmissions of opposite sample orientations, resulting from the significant synthetic moving effect of the metamaterial. The corresponding simulation result is shown in Fig. 4c, d, showing good agreement with the measured results. Therefore, the wavefront characterization further confirms the moving response of the metamaterial.

### Polarization-independent isolators

Compared with other passive‑system mechanisms for generating nonreciprocal phenomena (see Table I in the Supplementary Materials), our moving metamaterials offer polarization‑independent nonreciprocity, facilitating the development of versatile photonic components such as gyrators and isolators. Here we discuss the design of the isolator with asymmetric transmission amplitude for two opposite propagation directions based on the moving metamaterial and a conventional dielectric material. To achieve asymmetric transmission amplitude, it is necessary to introduce material loss into the system or incorporate additional diffraction orders to increase the number of channels in the scattering matrix. However, the inclusion of material loss significantly reduces transmission. Therefore, here we elaborate on the design of a polarization-independent isolator using a combination of moving metamaterial and a normal dielectric material, achieved through the introduction of additional diffraction orders.

**Fig. 5:**
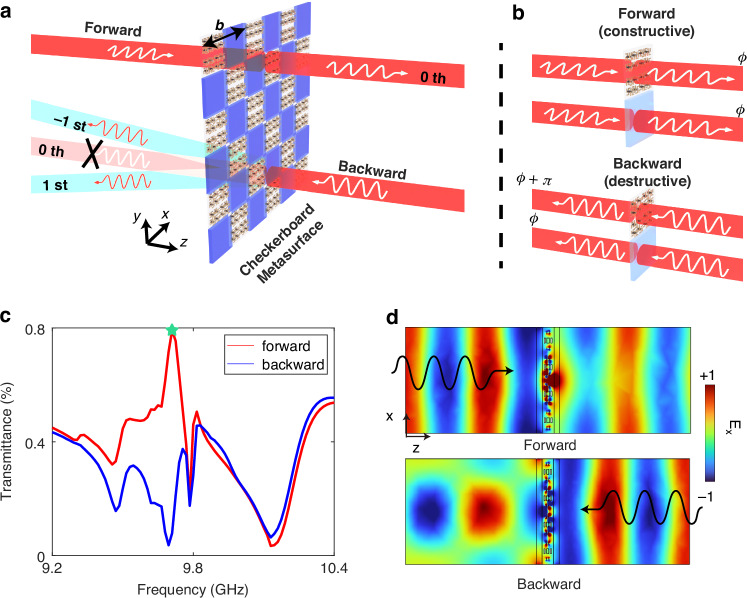
A polarization-independent isolator. **a** Schematic of the polarization-independent isolator: a checkboard metasurface combined with the moving metamaterial and the conventional dielectric material. The supercell comprises a moving medium containing four moving metamaterial unit-cells and an equivalent area of dielectric medium, with the periodicity b = 59.2 mm. **b** The mechanism enabling one-way transmission, involving constructive interference for forward incidence (upper panel) and destructive interference for backward incidence (lower panel). **c** Simulated transmittance of the isolator for forward and backward incidence. **d** Field distributions of *E*_*x*_ at the frequency of 9.7 GHz for forward and backward incidence

As illustrated in Fig. 5a, a checkerboard metasurface, implemented as a two‑dimensional grating with an alternating arrangement of a moving metamaterial and a normal dielectric material, enables the exclusive transmission of waves in one direction. Specifically, the incident wave’s power is transmitted through the zeroth diffraction order in the forward direction. However, for the backward propagation, the power is steered towards ± 1 orders. The underlying mechanism is schematically illustrated in Fig. 5b. By adjusting the structure parameters of the moving metamaterial, we can achieve a π phase difference between the forward and backward transmission, accompanied by a high transmission amplitude. By combining a dielectric material that matches the transmission phase of the moving metamaterial in the forward direction, the light transmitting through the moving metamaterial region constructively interferes with that of the dielectric region in the forward direction (upper panel in Fig. 5b). However, in the backward direction, the π phase shift of the moving medium induces destructive interference, resulting in minimal transmission through the zeroth diffraction order while steering towards the 1st order (lower panel in Fig. 5b).

For implementation, the unit‑cell geometry of the moving metamaterial is adjusted to produce a π phase difference between forward and backward transmission, functioning as a gyrator (see Supplementary Materials Sec. 10). The moving metamaterial, composed of four unit-cells, is alternated with a normal dielectric material to form a two‑dimensional checkerboard grating, as shown in Fig. 5b. Each square in the checkerboard has a side length of 29.6 mm, and the normal dielectric is a PCB slab with a thickness of 6 mm. The one-way transmission of the checkerboard metasurface is substantiated by the simulated transmittance, depicted in Fig. 5c. We noted that transmittance is defined as the intensity ratio of the transmitted zeroth-order wave and the incident wave. The forward transmittance (red curve) exhibits peak values at 9.7 GHz, aligning with the frequency of the moving medium featuring a π phase difference. In the backward direction, transmittance diminishes to near-zero levels due to destructive interference. Beyond the designated frequency, the phase difference decreases, resulting in comparable transmittance levels on both sides. The one-way transmission is further supported by the field distribution at 9.7 GHz in Fig. 5d. In the forward direction, the transmitted wave remains nearly a plane wave, confirming that nearly all the energy is directed into the zeroth order. In contrast, the transmitted light primarily deflects to 1st order in the backward direction. As a result, a polarization-independent isolator is realized using an all-passive, linear architecture.

## Discussion

In summary, we have experimentally demonstrated a striking moving response, corresponding to a large in-phase magnetoelectric coupling, in a static metamaterial operating in the microwave range. The metamaterial design incorporates locally combined gyromagnetic and chiral responses to achieve the intended moving effect, while being globally reconfigured to cancel out the parasitic gyromagnetic and chiral responses. This has led to the observation of a moving response with an effective moving velocity approaching 0.3c. It is noteworthy that, although the synthetic moving metamaterial shares the same form of constitutive relations as a real moving medium, there are inherent differences between them. For our metamaterial, we only consider monochromatic incident waves without the Doppler effect. The metamaterial is designed to achieve the cross-coupling between electric and magnetic fields in a manner analogous to that of a non-dispersive real moving medium at each frequency. With such constitutive parameters, the metamaterial can emulate the electromagnetic phenomena observed in a real moving magnetodielectric medium, thereby introducing a phase difference between forward and backward propagating waves. However, when considering dispersion, our synthetic moving metamaterial and a real moving medium would show different electromagnetic behaviors^36–38^. Nonetheless, our study carries significant implications for extending the capability of metamaterials beyond natural limitations. In addition, the synthetic moving metamaterials enable asymmetric transmission and facilitate the design of polarization‑independent isolators within an all‑passive, linear architecture, offering substantial advantages for practical applications.

## Methods

### Simulation

We simulated the transmission and reflection spectra of the device using the commercial software COMSOL Multiphysics based on the finite element method (FEM). The relative permeability tensor of the gyromagnetic materials has the form $$\tilde{\mu }=\left[\begin{array}{ccc}{\mu }_{r} & i\gamma & 0\\ -i\gamma & {\mu }_{r} & 0\\ 0 & 0 & 1\end{array}\right]$$, where ${\mu }_{r}=1+\frac{\left({\omega }_{0}+i\alpha \omega \right){\omega }_{m}}{{\left({\omega }_{0}+i\alpha \omega \right)}^{2}-{\omega }^{2}},\gamma =\frac{\omega {\omega }_{m}}{{\left({\omega }_{0}+i\alpha \omega \right)}^{2}-{\omega }^{2}},{\omega }_{m}=4{\rm{\pi }}\rho {M}_{s},{\omega }_{0}=\rho {\mu }_{0}{H}_{0}$, and *μ*_0_ *H*_0_ is the external magnetic field along the *z* direction which is considered as 0.16 T, *ρ* = 2.8 MHz/Oe is the gyromagnetic ratio, α = 0.001 is the damping coefficient, and *ω* is the operating frequency. The saturation magnetization has been set to 4π*M*_*s*_ = 1780 Oe. Permanent magnets are modeled as perfect electric conductors in simulations. Non-zero insertion loss is used in the simulation (PCB material) to match the practical experiment. Since the metasurface is periodic, we simulate a single unit cell with periodic boundary condition.

### Experimental setups

The schematic diagram of the experimental setup is shown in Supplementary Fig. S13. The sample is placed horizontally between the source and the probe. A horn antenna is placed under the sample, acting as a source to generate a nearly plane wave with y-polarization. A probe is placed along the y-axis above the sample to measure the *E*_*y*_ component of field. The dipole antenna is a coaxial cable with a 5 mm length of the outer conductor, leaving the central conductor exposed. This setup facilitates the measurement of both the amplitude and the phase of the field parallel to its orientation. Subsequently, the dipole antenna is driven by a three-axis translation stage to scan the field distribution. To measure the transmission and reflection spectra, two identical rectangular linearly polarized wideband horn antennas are employed to perform a frequency sweep in the range of 8 to 15 GHz.

## Supplementary information

Supplementary Materials for Observation of Synthetic Moving Effect in Metamaterials

## Data Availability

Data supporting key conclusions are available in the main text and the Supplementary materials. Data that supports all other findings of this study are available from the corresponding author upon request.
