# Supplementary material for: Observation of synthetic moving effect in metamaterials
Source: Light Sci Appl. 2026 Jun 8;15:268. doi: 10.1038/s41377-026-02361-y (PMC13243441; doi:10.1038/s41377-026-02361-y)
Supplement: Supplementary file 1 — Supplementary Materials for Observation of Synthetic Moving Effect in Metamaterials [file 41377_2026_2361_MOESM1_ESM.docx]

Supplementary Information for

**Observation of Synthetic Moving Effect in Metamaterials**

Qingdong Yang^1^, Zhongfu Li^1^, Xinhua Wen^1^, Oubo You^1^, Teruya Ishihara^1^, Xiaobo Yin^2^, Shuang Zhang^1,3,4,5,6^

**Affiliations:**

^1^ New Cornerstone Science Laboratory, Department of Physics, University of Hong Kong; 999077, Hong Kong, China

^2^ Department of Mechanical Engineering, University of Hong Kong; 999077, Hong Kong, China

^3^ State Key Laboratory of Optical Quantum Materials, University of Hong Kong; 999077, Hong Kong, China

^4^ Materials Innovation Institute for Life Sciences and Energy (MILES), HKU-SIRI, Shenzhen, China

^5^ [Quantum Science Center of Guangdong-Hong Kong-Macao Great Bay Area](https://ror.org/03qb6k992), 3 Binlang Road, Shenzhen, China

^6^ Department of Electrical and Electronic Engineering, [The University of Hong Kong](https://ror.org/02zhqgq86), Hong Kong 999077, China

1. Derivation of eigenmodes and the transmission of the slab with moving coupling

For an electromagnetic wave propagating along the z direction, the effective electromagnetic response of the moving medium can be written as the following constituent equations:

$\left( \begin{matrix} D_{x} \\ D_{y} \end{matrix} \right)=\varepsilon_{0}\varepsilon\left( \begin{matrix} E_{x} \\ E_{y} \end{matrix} \right)+\frac{1}{c}\left( \begin{matrix} 0 & -\xi\\ \xi& 0 \end{matrix} \right)\left( \begin{matrix} H_{x} \\ H_{y} \end{matrix} \right),$

$\left( \begin{matrix} B_{x} \\ B_{y} \end{matrix} \right)=\frac{1}{c}\left( \begin{matrix} 0 & \xi\\ -\xi& 0 \end{matrix} \right)\left( \begin{matrix} E_{x} \\ E_{y} \end{matrix} \right)+\mu_{0}\mu\left( \begin{matrix} H_{x} \\ H_{y} \end{matrix} \right).$ (S1)

According to the Maxwell's equations, Eq. S1 can be rewritten as a matrix form with the fields organized into a four-vector $\boldsymbol{G}=\left( E_{x},E_{y},\eta_{0}H_{x},\eta_{0}H_{y} \right)^{T}$, where $\eta_{0}=\sqrt{\mu_{0}/\epsilon_{0}}$ is the free-space impedance:

$\left( \begin{matrix} \mathbf{0} & i\boldsymbol{\nabla}\times\\ -i\boldsymbol{\nabla}\times& \mathbf{0} \end{matrix} \right)\boldsymbol{G}=k_{0}\boldsymbol{MG}$ (S2)

where ***M***=$\left( \begin{matrix} \begin{matrix} \varepsilon& 0 \\ 0 & \varepsilon\end{matrix} & \begin{matrix} 0 & -\xi\\ \xi& 0 \end{matrix} \\ \begin{matrix} 0 & \xi\\ -\xi& 0 \end{matrix} & \begin{matrix} \mu& 0 \\ 0 & \mu\end{matrix} \end{matrix} \right).$The above equation can be rewritten into the Hamiltonian form commonly used in quantum mechanics^1^:

$\boldsymbol{HG}=k_{0}\boldsymbol{G},$ (S3)

where ${\boldsymbol{H}=\boldsymbol{M}}^{-1}\left( \begin{matrix} \mathbf{0} & i\boldsymbol{\nabla}\times\\ -i\boldsymbol{\nabla}\times& \mathbf{0} \end{matrix} \right)$. Then we can calculate the eigenmodes of the medium for a plane wave. For the eigenmodes in the forward direction, the corresponding effective refractive index is $n_{\mathrm{fw}}=\sqrt{\varepsilon}\sqrt{\mu}-\xi$, with the corresponding linearly polarized eigenstates $\left( E_{x},E_{y},\eta_{0}H_{x},\eta_{0}H_{y} \right)^{T}$:$\boldsymbol{\phi}_{\mathrm{fw}}^{(1)}=\begin{aligned} & \\ & \\ & \\ & \end{aligned}\left( \frac{\sqrt{\mu}}{\sqrt{\varepsilon}},0,0,1 \right)^{T}, \boldsymbol{\phi}_{\mathrm{fw}}^{(2)}=\left( 0,-\frac{\sqrt{\mu}}{\sqrt{\varepsilon}},1,0 \right)^{T}.$For the backward direction, the effective refractive index is $n_{\mathrm{bw}}=\sqrt{\varepsilon}\sqrt{\mu}+\xi$, with the corresponding eigenstates:$\begin{aligned} & \\ & \\ & \\ & \end{aligned} \boldsymbol{\phi}_{bw}^{(1)}=\left( -\frac{\sqrt{\mu}}{\sqrt{\varepsilon}},0,0,1 \right)^{T},\boldsymbol{\phi}_{\mathrm{bw}}^{(2)}=\left( 0,\frac{\sqrt{\mu}}{\sqrt{\varepsilon}},1,0 \right)^{T}$.

1. Resonance behavior of the conjugated gammadion

When the electric field is incident upon the structure, current is induced in the upper and lower metal layers, forming an effective loop, as shown in Fig. S1a, generating a magnetic dipole viewed as chiral response which is the same direction as the electric field. The resonance behavior of the conjugated gammadion structure facilitates a strong confinement of the magnetic field within the gap between the two layers, as shown in Fig. S1b. As a result, the incident field can efficiently interact with the YIG material placed inside the gap, resulting in a notably enhanced bianisotropic moving response, which correlates with the observed large $v/c$ in the practical scenarios with a reasonable refractive index.

1. Symmetry based argument to remove other response of the unit cell

When the metamaterial structure respects the mirror symmetry with respect to the x/y axis (M_x_/M_y_) and parity–time (PT) symmetry, it can only possess the moving response out of all the bianisotropic responses. Here we provide symmetry analysis based on the Hamiltonian of the medium. For better analyzing the spatial symmetry, the Hamiltonian obtained in Section 1 needs to be expanded to a $6\times6$ matrix with the fields in three-dimensional space. As the designed structure holds the C4 symmetry in *x-y* plane, we consider following in-plane isotropic material parameters as the general constituent equations^1^:

$\boldsymbol{\varepsilon}=\left( \begin{matrix} \varepsilon& 0 & 0 \\ 0 & \varepsilon& 0 \\ 0 & 0 & \varepsilon\end{matrix} \right), \boldsymbol{\xi}=\left( \begin{matrix} i\kappa+\tau& i\Omega-V & 0 \\ -i\Omega+V & i\kappa+\tau& 0 \\ 0 & 0 & 0 \end{matrix} \right), \boldsymbol{\mu}=\left( -\begin{matrix} \mu& i\gamma& 0 \\ i\gamma& \mu& 0 \\ 0 & 0 & \mu\end{matrix} \right)$ (S4)

It includes four types of bianisotropic coupling: chiral coupling $i\kappa$, Omega coupling $i\Omega$, Tellegen coupling$\tau$ and moving coupling $V$, as well as the gyromagnetic effect $i\gamma$ generated by the YIG. If the structure possesses certain symmetry, the Hamiltonian must be satisfied:

$M\left( D_{i} \right)H(\boldsymbol{r})\left[ M\left( D_{i} \right) \right]^{-1}=H\left( D_{i}\boldsymbol{r} \right),$ (S5)

where $D_{i}$ is the symmetry operation, $M\left( D_{i} \right)$ denotes its matrix representation at $G$ vector space, and $\boldsymbol{r}$ is position vector.

If the system obeys M_x_ mirror symmetry, the corresponding operation would be $M\left( D_{i} \right)=diag\left( -1,1,1,1,-1,-1 \right);D_{i}=diag\left( -1,1,1 \right).$ If it further obeys M_y_ mirror symmetry, the corresponding operation would be $M\left( D_{i} \right)=diag\left( 1,-1,1,-1,1,-1 \right);D_{i}=diag\left( 1,-1,1 \right).$ After some simple derivation, in order to obey M_x_ and M_y_ mirror symmetry requirement for the Hamiltonian, we must have $\kappa=0$, $\tau=0, \gamma=0.$

When the system obeys PT symmetry, the corresponding operation is $M\left( D_{i} \right)=diag\left( -1,-1,-1,-1,-1,-1 \right)K, D_{i}=diag\left( -1,-1,-1 \right)$. K is the complex conjunction operation. It is straightforward to show that the PT symmetry enforces $\Omega=0,\gamma=0,\kappa=0.$

Hence, when the system satisfies M_x_ and M_y_ mirror symmetry and PT symmetry, the bianisotropic tensor $\xi$ can be simply written as,

$\xi=\left( \begin{matrix} 0 & -V & 0 \\ V & 0 & 0 \\ 0 & 0 & 0 \end{matrix} \right)$ (S6)

which only contains moving coupling terms.

1. An alternative design to realize the pure moving response

In the main text, we have demonstrated the moving response by combining the gyromagnetic response with the chiral response. Alternatively, the moving response can be realized by integrating the gyromagnetic response and the Omega response. Here we propose an alternative bianisotropic moving metamaterial design combining gyromagnetic materials (GM) and Omega structures. The proposed structure, as shown in Fig. S2, comprises an Omega structure and a GM biased by a static magnetic field $B_{y}^{0}$ in the *y*-direction. Similarly, as the wave impinges on the biased GM, the magnetic field $H_{x}$ induces a magnetic dipole moment $-{im}_{z}$. Consequently, the magnetic dipole field induces an electric dipole in the Omega structure along the *y*-direction. By combining the $-\frac{\pi}{2}$ phase delay from gyromagnetic effect and $\frac{\pi}{2}$ phase delay caused by Omega structures, this structure generates an in-phase electric dipole $p_{y}$, representing the moving response as discussed in the main text.

The designed metamaterial supercell is shown in Fig. S3. This supercell consists of four similar elements shown in Fig. S2. Each element consists of a metallic split ring resonator (gold color), and a YIG rod (black) sandwiched between two permanent magnets (silver). The overall unit cell exhibits C4 rotational symmetry. The blue arrow represents the direction of the static magnetic field. This configuration effectively cancels out unwanted responses, including gyromagnetic and Omega responses, leading to a pronounced pure moving effect for cases with a negligible interaction between the sub-units. For this metamaterial, the simulated transmission and reflection spectra are shown in Fig. S4. Similarly, a phase difference between the forward and backward transmitted waves can be observed, as indicated by the red curve in Fig. S4a. However, the transmission amplitude and reflection coefficients remain identical for waves incident from opposite directions. Furthermore, no cross-polarization is detected within the frequency range of interest, indicating a bianisotropic moving response.

1. Dipole moments induced by the moving response

Here we provide more details about the electric and magnetic dipoles moments induced by the moving response. We have presented two approaches for designing moving metamaterials. The first approach involves the combination of the gyromagnetic materials and chiral structures, as depicted in Fig. 1b in the main text. The second approach combines the gyromagnetic materials and Omega structures, as shown in Fig. S2 in Section 4.

For the first approach, a gyromagnetic material biased by an external magnetic field along the z-direction, the relative permeability tensor has the form of $\left( \begin{matrix} \mu& i\gamma& 0 \\ -i\gamma& \mu& 0 \\ 0 & 0 & \mu\end{matrix} \right)$. When an x-oriented magnetic field $H_{x}$ impinges on the gyromagnetic material, a dipole moment $-{im}_{y}$ is generated through the gyromagnetic effect (i.e., the antisymmetric elements of the permeability tensor), i.e.,

$$\left( \begin{matrix} \mu& i\gamma& 0 \\ -i\gamma& \mu& 0 \\ 0 & 0 & \mu\end{matrix} \right)\left( \begin{matrix} H_{x} \\ 0 \\ 0 \end{matrix} \right)\to-{im}_{y}$$

Here the factor $-i$ indicates that the induced magnetic dipoles have a phase shift of $-\pi/2$ with respect to the magnetic field. Combining the chiral effect $i\kappa$, the magnetic dipole $-{im}_{y}$ transforms into an electric dipole $p_{y}$. Specifically, the time-varying magnetic dipole $-{im}_{y}$ induces an electric current in the chiral structures, resulting in accumulated charges of opposite charges at two ends and forming an electric dipole $p_{y}$ along the y-direction. Therefore, by combining the $-\frac{\pi}{2}$ phase delay from the gyromagnetic effect and the $\frac{\pi}{2}$ phase delay caused by chiral structures, the resulting electric dipole $p_{y}$ is in phase with the incident magnetic field $H_{x}$, exactly achieving the desired moving response.

For the second approach depicted in Fig. S1, a gyromagnetic material biased by an external magnetic field along the y-direction, the relative permeability tensor has the form of $\left( \begin{matrix} \mu& 0 & i\gamma\\ 0 & \mu& 0 \\ -i\gamma& 0 & \mu\end{matrix} \right)$. For the incident magnetic field $H_{x}$, a dipole moment $-{im}_{z}$ is generated by

$$\left( \begin{matrix} \mu& 0 & i\gamma\\ 0 & \mu& 0 \\ -i\gamma& 0 & \mu\end{matrix} \right)\left( \begin{matrix} H_{x} \\ 0 \\ 0 \end{matrix} \right)\to-im_{z}$$

Similarly, the induced magnetic dipoles have a phase shift of $-\pi/2$ with respect to the magnetic field. Combining the Omega effect $i\Omega$, an electric dipole $p_{y}$ is generated, indicating the realization of the moving response. Therefore, the nonreciprocal moving coupling, as well as Tellegen coupling, characterized by real coupling coefficients, provide the in-phase cross-coupling between the electric and magnetic response. In contrast, for reciprocal chiral and Omega coupling characterized by imaginary coupling coefficients, the induced dipole moment has an additional π/2 phase shift with respect to incident waves.

1. The fabricated sample

The fabricated sample is around the size of $40cm\times40cm\times3mm$ on the left side of Fig. S5a. The unit cell (a=14.8 mm) is composed of five distinct layers, as shown on the right side of Fig. S5a. In the 5th (top) layer, the arrangement of the magnets is the same as the 1st layer which is shown in Fig.2b in main text, generating a strong static magnetic field in the 3rd (middle) layer where YIG rods are located. In the 2nd layer, four gammadions are arranged as designed. The arrangement of the 4th layer is conjugated gammadion structure. The detailed parameters of the unit-cell are shown in Fig. S5b. At the surface of the gammadion layers (with a thickness of $h_{1}$=0.25 mm), metallic structures are fabricated using traditional Printed Circuit Board (PCB) technology in Teflon. The gammadion structure holds C4 symmetry, with the dimensions of the arms of the structure noted as $L_{1}$=2.1mm and $L_{2}$=3.2 mm, and a width of w=0.38 mm. The YIG layer with a thickness of $h_{2}=1$mm, consists of YIG cylinders with a diameter of d=2 mm and a height of 1mm. The permanent magnet layers, with a thickness of $h_{3}$=0.5mm, incorporate permanent magnets with a diameter of d=2 mm and a height of 0.5 mm. Both the permanent magnets and the YIG rods are embedded within the Teflon slab of each layer. Due to their small physical size (approximately 1/15 of the operating wavelength) and the absence of any resonance behavior in the frequency range of interest, the magnets have a negligible impact on the transmission characteristics of the metamaterial.

1. A moving metamaterial with broadband chiral structures

In the current design, the enhanced moving effect arises from the resonance behavior of the chiral metallic elements (as illustrated in Fig. S1), which inherently limits the transmission to a relatively narrow bandwidth. To enable broadband operation, we investigated a modified chiral structure with broadband chiral response based on the Huygens’ metasurface principle ^2^. The proposed unit cell, shown in Fig. S7a, has a slab thickness of 3 mm (approximately one-tenth of the operating wavelength) and preserves the configuration and symmetry of the structure described in the main text. As illustrated in Fig. S7b, simulations reveal a transmittance exceeding 90% over a 1.5 GHz range and above 50% over a 3 GHz range. Importantly, a pronounced phase difference between forward and backward transmitted waves is maintained throughout the broadband frequency range.

1. Derivation of transmission and effective velocity

In Section 1, we obtain the effective refractive index $n_{\mathrm{fw}}$ ($n_{\mathrm{bw}}$) and eigenstates $\boldsymbol{\phi}_{\mathrm{fw}}^{(1)},\boldsymbol{\phi}_{\mathrm{fw}}^{(2)}$ ($\boldsymbol{\phi}_{\mathrm{bw}}^{(1)},\boldsymbol{\phi}_{\mathrm{bw}}^{(2)}$) for forward (backward) propagating waves within the bianisotropic moving medium, then we can calculate the transmission and reflection coefficients based on the continuous boundary conditions. As shown in Fig. S8, when the forward propagating light is incident onto the first interface, part of the incident light is reflected, and the rest gets transmitted. Applying the continuous boundary condition of the tangential fields at the first interface, we can get

$$\begin{aligned} \boldsymbol{F}^{\mathrm{in}}+a\boldsymbol{F}_{\mathrm{co}}^{R}+b\boldsymbol{F}_{\mathrm{cr}}^{R}=c\boldsymbol{\phi}_{\mathrm{fw}}^{\left( 1 \right)}+d\boldsymbol{\phi}_{\mathrm{fw}}^{\left( 2 \right)}+e\boldsymbol{\phi}_{\mathrm{bw}}^{\left( 1 \right)}+f\boldsymbol{\phi}_{\mathrm{bw}}^{\left( 2 \right)}, \#\left( S7 \right) \end{aligned}$$

where $\boldsymbol{F}^{\mathrm{in}}$ represents the incident wave with a unit amplitude. $\boldsymbol{F}_{\mathrm{co}}^{R}$ and $\boldsymbol{F}_{\mathrm{cr}}^{R}$ respectively represent the co-polarized and cross-polarized reflected waves, $a$ and $b$ represent the corresponding coefficients. $c,d,e,$ and $f$ represent the coefficients of four eigenmodes of the moving metamaterial.

Similarly, applying the continuous boundary condition at the second interface,

$$\begin{aligned} c\boldsymbol{\phi}_{\mathrm{fw}}^{\left( 1 \right)}e^{i{n_{\mathrm{fw}}k}_{0}d_{0}}+d\boldsymbol{\phi}_{\mathrm{fw}}^{\left( 2 \right)}e^{i{n_{\mathrm{fw}}k}_{0}d_{0}}+e\boldsymbol{\phi}_{\mathrm{bw}}^{\left( 1 \right)}e^{i{n_{\mathrm{bw}}k}_{0}d_{0}}+f\boldsymbol{\phi}_{\mathrm{bw}}^{\left( 2 \right)}e^{i{n_{\mathrm{bw}}k}_{0}d_{0}}=g\boldsymbol{F}_{co}^{T}+h\boldsymbol{F}_{cr}^{T},\#(S8) \end{aligned}$$

where $\boldsymbol{F}_{\mathrm{co}}^{T}$ and $\boldsymbol{F}_{\mathrm{cr}}^{T}$ respectively represent the co-polarized and cross-polarized transmitted waves, $g$ and $h$ represent the corresponding coefficients. $k_{0}$ is the vacuum wavevector and $d_{0}$ is the effective thickness of the slab. Combining the two sets of equations Eq. S8 and Eq. S9 we can get the reflection ($a,b$) and transmission ($g,h$) coefficients for the forward incidence. Similarly, the transmission and reflection coefficients for backward incidence can be calculated based on the continuous boundary conditions.

As a result, the Fresnel’ equation for the reflection and transmission can be obtained:

$$T_{\mathrm{co}}^{\pm}=\frac{4Zⅇ^{ⅈnk_{0}d_{0}}ⅇ^{\mpⅈk_{0}d_{0}\xi}}{\left( 1+Z \right)^{2}-ⅇ^{2ⅈnk_{0}d_{0}}\left( 1-Z \right)^{2}},$$

$$R_{\mathrm{co}}^{\pm}=\frac{\left( -1+ⅇ^{2ⅈnk_{0}d_{0}} \right)\left( 1-Z^{2} \right)}{\left( 1+Z \right)^{2}-ⅇ^{2ⅈnk_{0}d_{0}}\left( 1-Z \right)^{2}},$$

$$\begin{aligned} R_{\mathrm{cr}}^{\pm}=T_{cr}^{\pm}=0, \#\left( S9 \right) \end{aligned}$$

where$\mathrm{the}$superscript $\pm$ represent forward and backward incidence, the subscript “co” and “cr” represent the co-polarized and cross-polarized waves.

With Fresnel’s equations, we can extract the effective parameters through:

$$\frac{T_{\mathrm{co}}^{+}}{T_{\mathrm{co}}^{-}}=e^{-2ik_{0}d_{0}\xi}$$

$$\frac{\left( 1+R_{\mathrm{co}}^{+} \right)^{2}-T_{\mathrm{co}}^{+}T_{\mathrm{co}}^{-}}{\left( 1-R_{\mathrm{co}}^{+} \right)^{2}-T_{\mathrm{co}}^{+}T_{\mathrm{co}}^{-}}=Z^{2}$$

$$\begin{aligned} \frac{1}{T_{\mathrm{co}}^{+}}\left( 1-\frac{Z-1}{Z+1}R_{\mathrm{co}}^{+} \right)=e^{-i\left( n-\xi\right)k_{0}d_{0}}\#\left( S10 \right) \end{aligned}$$

Once the impedance and refractive index are obtained, the effective permittivity and permeability can be determined through $\epsilon=n/Z, \mu=nZ$.

After we get the effective parameters ($\varepsilon,\mu,\xi$), the effective velocity can be obtained based on the Lorentz transformations:

$$\begin{aligned} \epsilon=\epsilon^{'}\frac{1-\beta^{2}}{1-\beta^{2}n^{'2}}, \mu=\mu^{'}\frac{1-\beta^{2}}{1-\beta^{2}n^{'2}}, \xi=\beta\frac{n^{'2}-1}{1-\beta^{2}n^{'2}}, \#\left( S11 \right) \end{aligned}$$

where$\beta=\frac{v}{c_{0}} .$ Then we can get the effective velocity:

$$\begin{aligned} \beta=\frac{-1+\epsilon\mu+\xi^{2}\pm\sqrt{-4\xi^{2}+\left( 1-\epsilon\mu+\xi^{2} \right)^{2}}}{2\xi}\#\left( S12 \right) \end{aligned}$$

Therefore, the effective velocity is determined not only by the bianisotropic moving‑coupling parameter, but also by the refractive index of the medium. To choose the right value, we apply two conditions: 1) *|β| < 1*; 2) *β* is continuous across the entire spectrum. Finally, we obtain the effective velocity.

1. The effective velocity

Here we discuss the effective velocity which emulates the behavior of a real moving medium. According to the relativistic velocity addition law, the relation between the effective refractive index $n_{+}$ of the forward propagating wave in the laboratory frame and the refractive index $n'$ in the moving frame can be expressed as

$$\begin{aligned} n_{+}=\frac{n^{'}+\beta}{n^{'}\beta+1}\#\left( S13 \right) \end{aligned}$$

For a normal material with $n^{'}$>1, when $\beta$ increases from 0 to 1, $n_{+}$ decreases from $n^{'}$ to $1$. Therefore, we have ${n_{+}<n}^{'},$ representing that light is dragged by the motion of the medium. For some special materials with $n^{'}$<1, when $\beta$ increases from 0 to 1, $n_{+}$ increase from $n^{'}$ to $1$ $and then we have {n_{+}>n}^{'}$. The corresponding phase velocity of the wave decreases from ${c/n}^{'}$ to $c$ and the light is slowed down by the motion of the medium. At this case, one need to reverse the sign of $\beta$ to realize ${n_{+}<n}^{'}$.

As we see $\xi$ is always positive across the whole regime as shown in Fig. 3c in the main text, indicating ${n_{+}<n}^{'}$ across the whole regime. Thus, as shown in Fig. S10, at frequencies below the resonance frequency, the materials possess $n^{'}<1$, behaving like metals. To realize faster speed than the original light speed in the corresponding stationary medium, the velocity should be negative. For frequencies above the resonance frequency, the refractive index becomes $n^{'}>1$. When the velocity is positive, the speed of light is dragged by the movement, which is faster than the original one. The anomalous optical drag at different refractive index causes the inverted sign of velocity. Similar analysis can be applied to the wave propagating against the moving direction of the velocity.

In the manuscript, away from the peak of resonance, imaginary part of velocity is caused by the intrinsic loss of the medium. In simulation, we neglect all the loss in the systems and retrieve the effective velocity in Fig. S11b. We observe that in a narrow frequency range around the resonance, the velocity has an imaginary part. Away from the peak of resonance, the imaginary part is almost zero, indicating a real value velocity. At the same time, the velocity is still large enough to observe (~0.4c) for this lossless case.

1. The design of gyrators

Here we discuss the design of the gyrator with a $\pi$ phase difference between the forward and backward transmission, by adjusting the geometry of the unit-cell structure of the moving metamaterial. The thickness of PCB slab is 4.6mm with $\varepsilon$=2.45. The length L_1_=3.1mm and L_2_=2.2mm and all other parameters are the same as the original one in the main text, as shown in Fig. S12a. The simulated reflection and transmission of this new metamaterial are shown in Fig. S12b. A π phase difference is achieved at the frequency of 9.7 GHz, accompanied by a high transmission amplitude for both the forward and backward incidence. Therefore, this new moving metamaterial holds promise for potential applications as a gyrator, using purely passive materials.

**Figures**


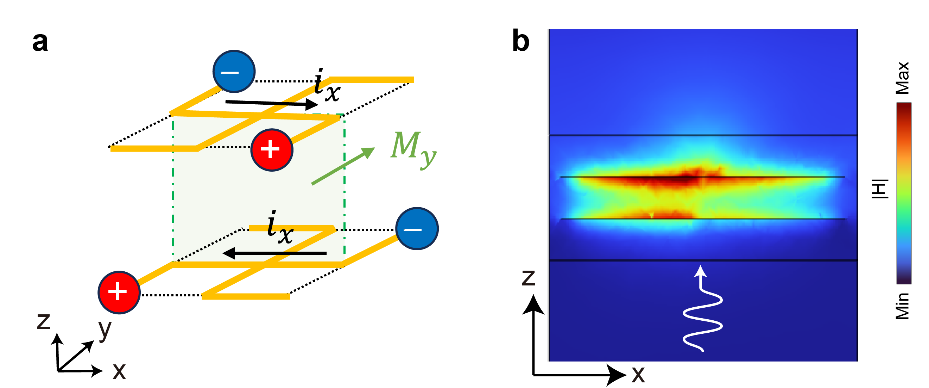


Fig. S1 The strong chiral response of conjugated gammadion structure when the Ey-polarized wave is incident along z axis. **a** The currents and accumulated charge in the chiral structure generate a strong chiral response. **b** The magnetic field distribution within the chiral unit cell at the frequency of 9.7 GHz shows a strong enhancement in the gap between two layers.


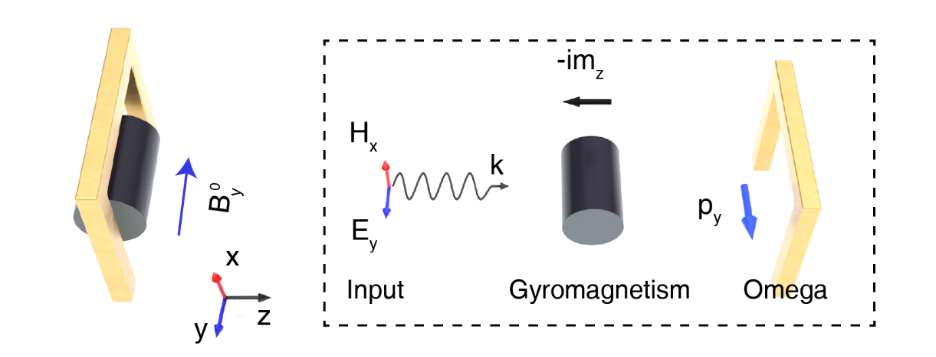


Fig. S2 An alternative design combined Omega and gyromagnetic effect to achieve the moving response. The x-oriented magnetic field $H_{x}$ induces a magnetic dipole ${-im}_{z}$ and then excites the electric current along the Omega structure inducing the electric dipole $p_{y}$.


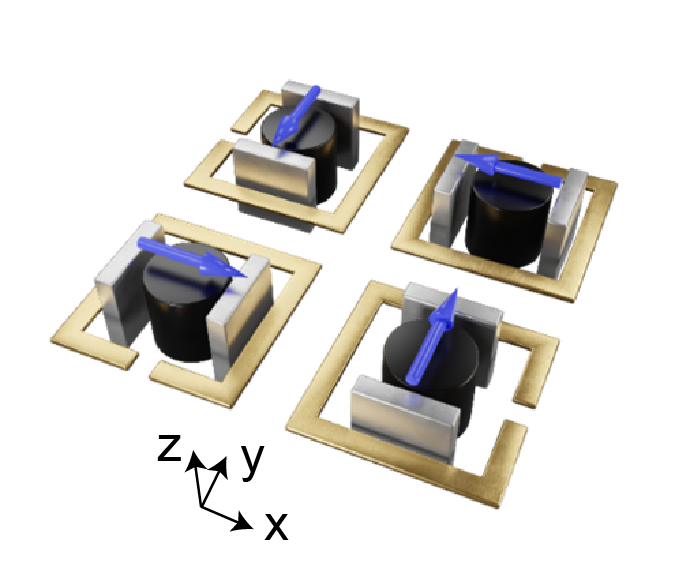


Fig.S3 The alternative metamaterial supercell for realizing the pure moving response. The supercell, with dimensions of $24 mm\times24 mm\times2mm$, consists of four sub-units biased by different external magnetic fields (blue arrows indicate the direction of bias magnetization). The length of the split-ring resonator in each sub-unit is L=8mm, and the permanent magnets (silver) have a dimension of $1mm\times4mm\times2mm$. The YIG cylinder (black) has a diameter of d=2 mm and a height of 2mm.


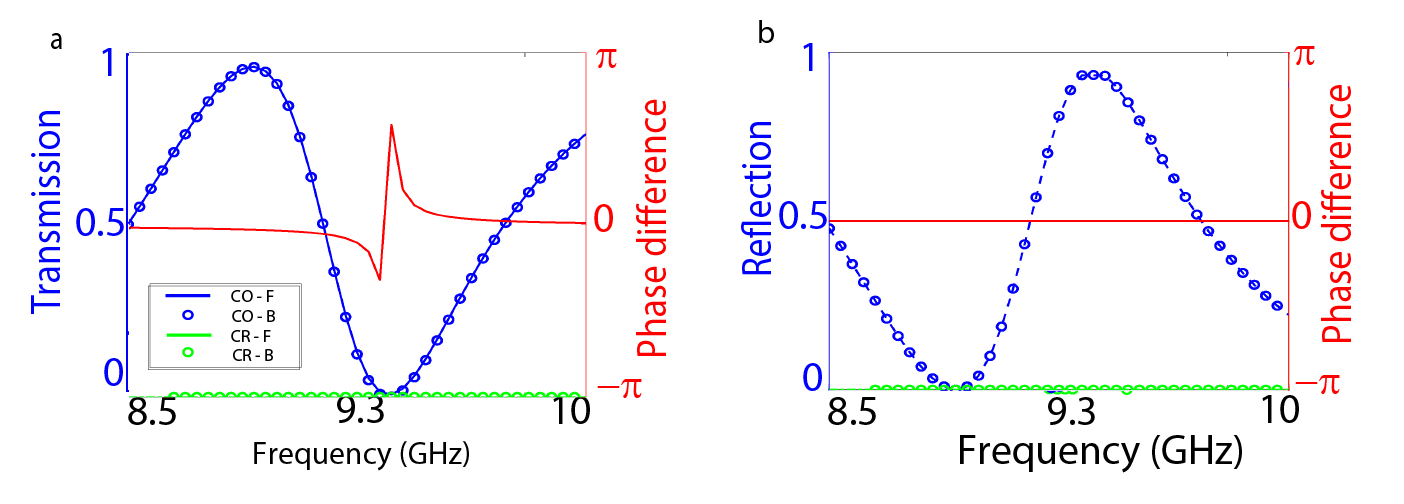


Fig.S4 Simulated results of the alternative bianisotropic moving metamaterial combined gyromagnetic materials and Omega structures. **a** Simulated transmission and **b** reflection spectra for an Ey-polarized incident wave. The labels “CO-” and “CR-” indicate co-polarization and cross-polarization respectively, and “F” and “B” represent forward and backward incident directions respectively.


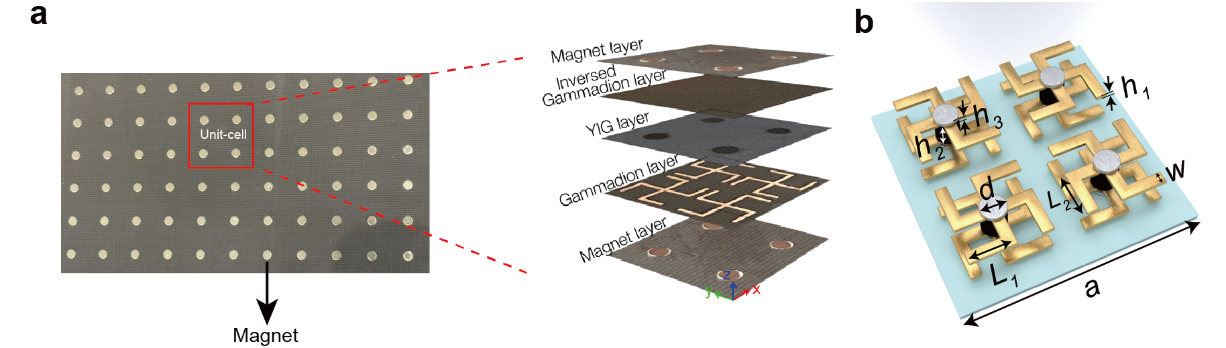


Fig. S5 **a** The photograph of a realistic sample consisting of 5 layers. **b** The geometry structure of the unit-cell.


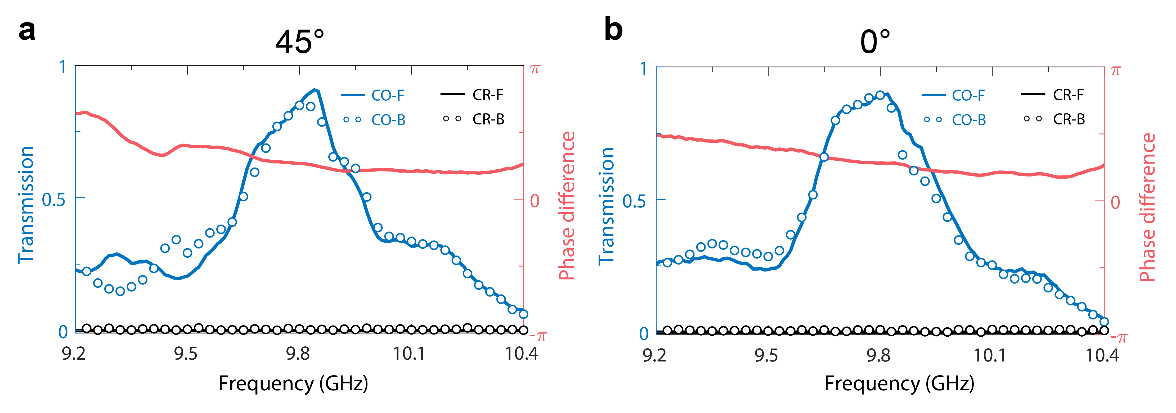


Fig. S6 Experimental results for different polarization incidence. Measured transmission and reflection spectra for linear polarized incident waves at **(a)** 45 degrees and **(b)** 0 degrees (x-polarized).


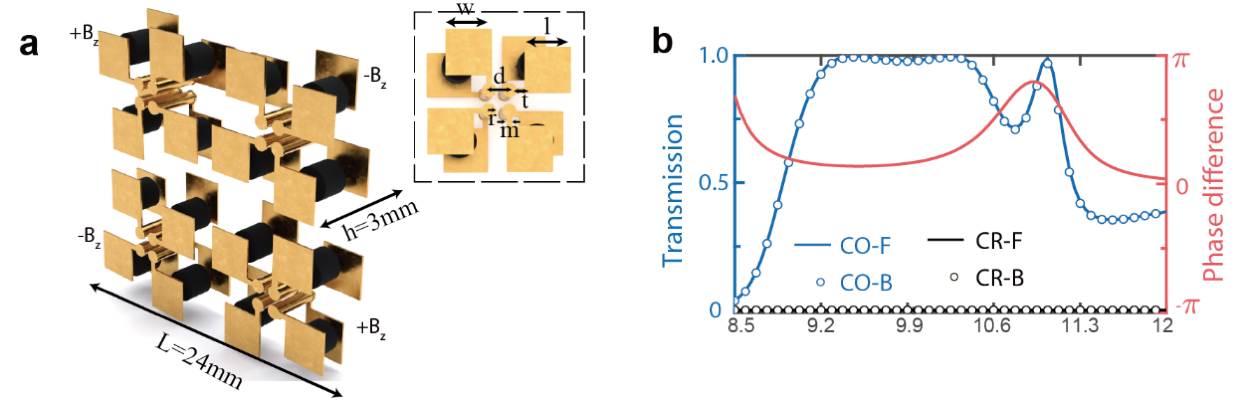


Fig.  S7 Modified broadband unit cell and simulated electromagnetic response. **a**, Schematic of the modified unit cell, consisting of four chiral metallic elements, each embedding a YIG rod (black cylinder). The applied static magnetic field follows the same configuration as in the main text. The structure has a periodicity of L=24 mm. Top view of one subunit cell is inserted to show geometrical parameters (in mm): r=0.45, d = 1.3, t =0.6, m=0.7, w= 2.4, l = 3.5. The slab permittivity is $\varepsilon_{r}=3.5$. **b**, Simulated transmission spectra for forward and backward incidence.


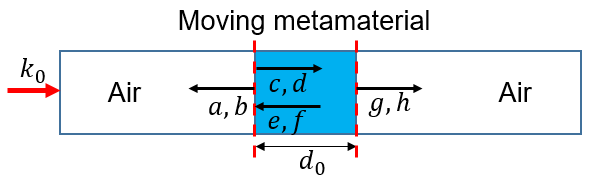


Fig. S8 The model for calculating the reflection and transmission of the metamaterial slab. The reflection and transmission of a moving metamaterial slab with a thickness of $d_{0}$, where $a,b$ and $g,h$ respectively represent the reflection coeffients and transmission coeffients of the co-polarized and cross-polarized waves, $c,d,e,$ and $f$ represent the coeffients of four eigenmodes of the moving metamaterial.


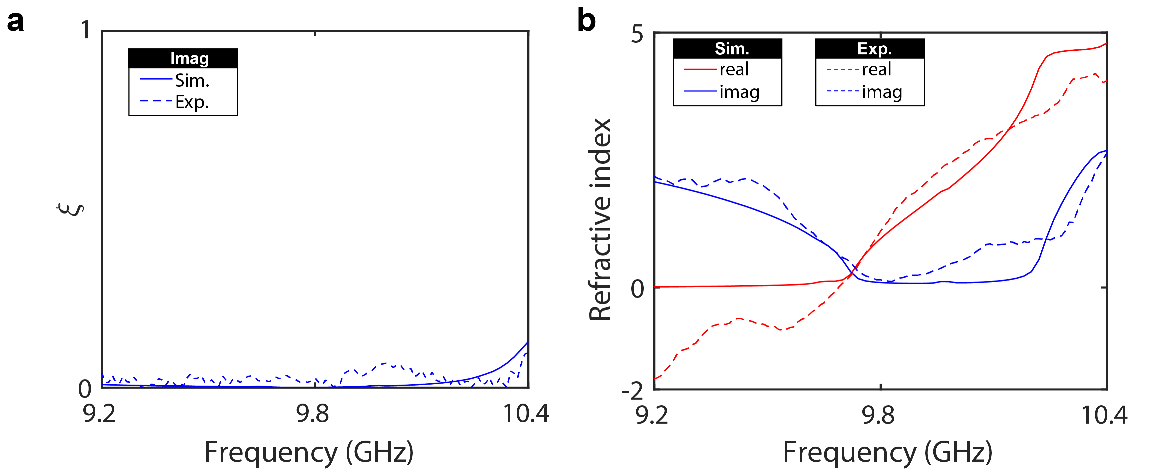


Fig. S9 The effective parameters extracted from the reflection and transmission spectrum. **a** The imaginary part of the effective bianisotropic moving coupling $\xi$. **b** The real (red curves) and imaginary parts (blue curves) of the refractive index $n$. The solid and dashed lines represent the simulated and measured results, respectively.


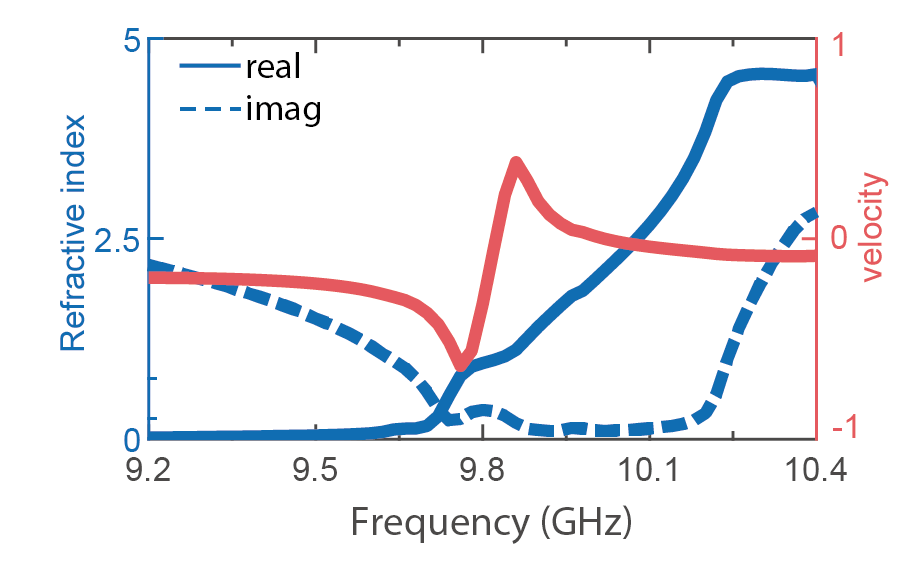


Fig.S10 The refractive index $n'$ of the medium in moving frame and effective velocity.


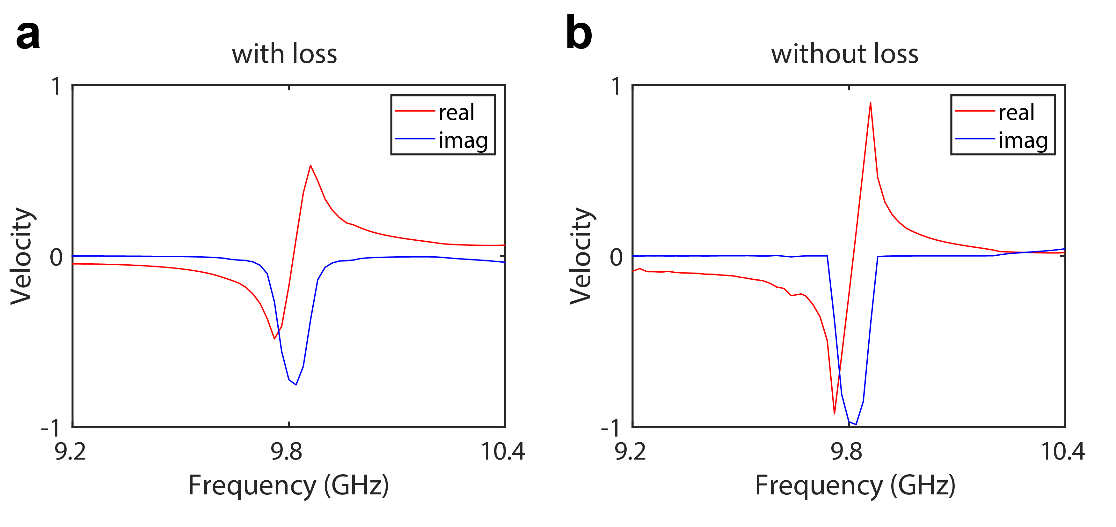


Fig.S11 The effective velocity of the effective medium in **(a)** loss and **(b)** lossless condition.


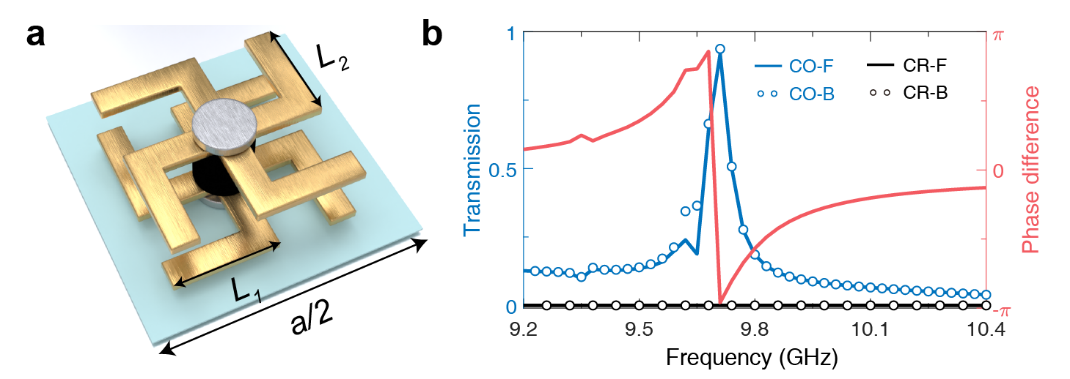


Fig. S12 The design of the gyrator. **(a)** Subunit cell of redesigned metamaterial for the gyrator. **(b)** The simulated transmission and reflection for forward and backward incidence. The red curve shows the phase difference from two opposite propagating waves.


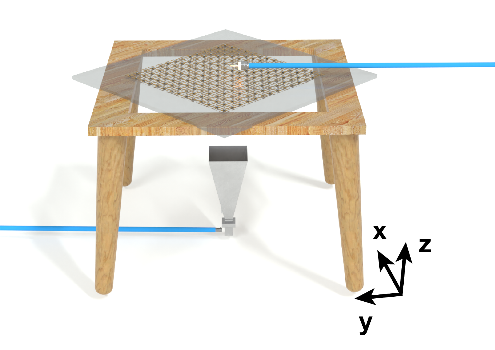


Fig. S13. Schematic of the near-field measurement setup.

**Table**

| Ref | Methods | Mechanism | Nonreciprocal feature | Polarization |
| --- | --- | --- | --- | --- |
| Nat. Electron., 6, 225–234 (2023) | Self-biased magnetic meta-atoms (La:BaM) | Gyrotropic response | Asymmetric phase and amplitude of transmission | circular polarization |
| Opt. Lett. 45, 5917-5920 (2020) | YIG materials | Gyrotropic response | Wave filter (Asymmetric intensity) | circular polarization |
| Nano Lett. 18, 1104–1109 (2018) | Silicon with pumping | Kerr nonlinearity | Asymmetric intensity | Arbitrary  polarization |
| Our work | YIG and chiral metal structure | bianisotropic moving response | Asymmetric phase and  same amplitude of transmission | Arbitrary  polarization |

Table I. Comparison of nonreciprocal metasurfaces.

**Reference**

1 Horsley, S. A. R. & Woolley, M. Zero-refractive-index materials and topological photonics. *Nat Phys* **17**, 348 (2021).

2 Li, J., Yuan, Y., Wu, Q., & Zhang, K. Bi-isotropic huygens’ metasurface for polarization-insensitive cross-polarization conversion and wavefront manipulation. *IEEE Trans. Antennas Propag.* **72**, 2445-2454 (2024).

3 Vehmas, J., Hrabar, S. & Tretyakov, S. Transmission lines emulating moving media. *New J Phys* **16,** 093065 (2014).
